# Supplementary material for: Living with Pain and Looking for a Safe Environment: A Qualitative Study among Nursing Students with Dysmenorrhea
Source: Int J Environ Res Public Health. 2020 Sep 13;17(18):6670. doi: 10.3390/ijerph17186670 (PMC7558082; doi:10.3390/ijerph17186670)
Supplement: Supplementary file 1 [file ijerph-17-06670-s001.pdf]

Supplementary Materials Table S1. Themes that emerged from the participant narratives

Theme: Living with dysmenorrhea.	
Subtheme: Menstruation	
Codes	Narratives
Menstruation perceived negatively: *"…It's hell for me, really."* (FG4); *"…it's horrible. I actually say the "menstruation."* (FG4); *"…awful, I mean... I just think about it and I feel ill."* (FG4); *"…for me it would be limiting and exhausting, because for me it is like the period is coming and I feel ugh (sigh) again, goodness me, it exhausts me."* (FG4).<br><br>Menstruation perceived as sickness: *"…we're from Ecuador, and my mother says I'm going to get sick with my period. Here it's 'you're going to get you're period,' but there it's 'you're going to get sick' to mean you're going to get your period."* (FG3); *"…My grandmother asks me if I'm already sick, instead of asking me whether I've got my period."* (FG3).<br><br>Concern, distress and shame: *"…I got my period during my internship and I stained my scrubs, how do I tell the nurses I was with that I got my period, that I stained my scrubs? I'm so embarrassed."* (FG1); *"a white uniform, it depends on the period, if it comes more abundant and stains it... if it happened to me, I would die of shame."* (FG3).<br><br>Difficulty sleeping: *"…I was up all night, and not only because of the pain, but also because of the insecurity wondering 'Did I stain my pajamas? Did I stain the sheets?'* (FG3); *"…I usually get my period at night... I get up with the pain, I go to the bathroom and I see that it has come... you are always worried that you might stain the pajamas, stain the sheet."* (FG3).<br><br>Social taboo of menstruation: *"…I think it's also taboo in society. I ask my classmates for a tampax or a pad, and it's typical that you keep it put it away like that so no one will see it."* (FG3); *"a friend said to me 'put away the pad' or 'you can see the pad there' to which I responded: 'well, if I carry it in my pocket it's because I'm going to the bathroom now, I'm not going to hide it as if it were something bad.'* (FG3).	
Subtheme: Pain.	
Codes	Narratives

Pain that is variable, intense and disabling: *"…the first days, especially the first, the pain limits me a lot." (FG1); "…the first day of your period, which is awful, because it's the one that stains the most, hurts the most and makes you want to do nothing at all." (FG2); "…if I am late, the pain is unbearable. I've been lying on the sofa for two days now." (FG4).*<br><br>Uncertainty, stress and fear: *"…afraid of the fact that, if I go out now, and it bothers me and I have to go home in five minutes. Then what will I do?"* (FG2); *"…it' s the uncertainty of going out, going anywhere and always taking pills, because I get dizzy too. It's fear of going out, going on public transport, being taken over by the pain and you start thinking 'what if I get dizzy? what if something else happens to me?'"* (FG2).	
Theme: Body and mood changes.	
Codes	Narratives
Bodily changes and self-perception: *"…I feel physically more deformed, because my clothes feel bad... my belly is super swollen, super hard."* (FG1); *"…I look in the mirror, and these are the days I look the most ugly. I say, 'How can I be like this?' I look at myself and I don't like it. These are the days of the month when I see myself as less attractive." (FG2); "…I don't have much of a belly, really, and when I get my period I get bloated. As if my normal belly tripled."* (FG2).<br><br>Changing how they dress: *"…the day I have my period and I have an event and I have to get ready, maybe put on some tights... it conditions me and bothers me and I look uglier because of it. You don't feel like getting dressed up."* (FG2). *"...when I've got my period, tight jeans and stuff like that I can't wear, I'd rather wear leggings or something comfortable."* (FG3).<br><br>Mood swings: *"…it's as if you feel like you're bipolar, sometimes you want a hug, sometimes you don't even want to be looked at …"* (FG5); *"…a few days before I already have ups and downs, mood swings, even during, there are times when I feel super well, and there are others when I don't even want to be spoken to... or because you feel depressed, sad, it affects you three times more."* (FG5).<br><br>Lack of energy and disruption of daily activities: *"…I don't feel so active, and there are a lot of things I could do, but I don't feel like I can do them, so I stop doing them. You have less energy."* (FG1); *"…it saps my energy to do some things."* (FG5)"; *"…for me it's more like body heaviness, like saying I lack the energy to do what I want."* (FG5).<br><br>Increased sensitivity/irritability with others: *"…I get very angry, even if I'm not right, but then I break down and I want to be loved, or I start crying because I want to be forgiven for having treated them badly."* (FG3); *"…everything is more on the surface, both the irritability, the fact that things bother you more, or that something that you do not give importance to on normal days, at that time you feel worse. Then the argument begins."* (FG2).	

Theme: Seeking a safe environment	
Subtheme: A safe environment	
Codes	Narratives
Feeling understood: *"…I usually feel that they understand me because I am surrounded by a circle of many women and most of them often have menstrual cramps and know what it is."* (FG1); *"…I have friends with dysmenorrhea, but it is true that we understand each other, because it is something natural, we know that there are days when we can be more irritable."* (FG4).<br><br>To be able to speak freely about dysmenorrhea: *"…I was with the nurses and it happened to be the day I started and I told them about it, or they noticed something and asked me 'is there something wrong with you?' and I told them it was because of the period."* (FG2).<br><br>People close to you see the consequences of pain: *"my friends didn't understand at first, but now they do, because they know me and they have experienced what the pain is like by living with me... I threw up and almost fainted, they were very scared."* (FG4); *"…when they experience it with you, let's say they understand you and they know it's real, that it's not an excuse or a coincidence, or something you're making up."* (FG4).	
Subtheme: An unsafe environment	
Codes	Narratives
When others disregard pain: *"…people who may not be in pain and as if I'm to blame for being in pain. They reproach you for it."* (FG4).<br><br>Women who understand it when they experience it *"…people don't understand it until they suffer from it. When they've lived through it, then they empathize more, they put themselves more in your shoes."* (FG4).<br><br>Women who haven't had a period in a long time *"…the environment I worked in, was with women who were mostly menopausal. It's like they lose their empathy, like they've forgotten that for them.* (FG2).<br><br>Unknown environments: *"…When you get to a new place, or maybe during the practice period and you have to do things, you aren't going to say 'Well, look, if you see that maybe I am not going so fast, do not demand much more', but I usually shut up, but if it' s on a day that they know you more, then you don't mind saying so."* (FG1).	
Subtheme: Key issues regarding security	

Codes	Narratives
A person close to you may or may not belong to the safe environment:	*"…on behalf of my family and my partner no, because they understand me, but my friends are like, 'Oh, jeez, are you gonna miss it?' Well, yes, because I can't."* (FG4).
Men may or may not understand dysmenorrhea/pain:	*"men usually understand me...they know that I am in pain, that I am more sensitive." (FG2); "...men think that you're exaggerating it, that it's nonsense, that you complain just for the sake of complaining."* (FG5); *"…I've felt understood by both men and women around me. Maybe some of them are more distant because they don't understand it, especially men, but I've felt understood by those around me."* (FG5).
Belonging to the safe environment is not a matter of gender, but of empathy:	*"…we may think they can think this of men, but it may also be thought of by women. There are women who have gone through this pain and have pushed through it, and they tell you that it's not an excuse... I think it's more a question of empathy, of people putting themselves in your place."* (FG2); *"…that depends more on the person than on the gender itself." (FG5*).
Concealing it in the practice/work environment:	*"…I tell him I'm sick due to another reason, or even tell him I have another problem." (FG2);"… If I had a very painful period that made me unable to go to work that day, I wouldn't say it was because of my period, because he' s going to say that because I'm a woman and I'm going to get my period often, it's better for us to get rid of it than to keep it here." (FG2); "…sometimes they think it's an excuse not to go to work, sometimes they say to you... 'What a coincidence!'" (FG4)*

Supplementary Materials Table S2. Themes, subthemes, and codes that emerged from Focus Groups

Theme	Subtheme	Codes	Focus group (FG)					Total
			FG1	FG2	FG3	FG4	FG5	
Living with dysmenorrhea	Menstruation	Menstruation perceived negatively	1	3	1	5	4	14
		Menstruation perceived as sickness	6	8	6	6	5	31
		Concern, distress and shame	3	1	16	1	1	22
		Difficulty sleeping	1	6	6	1	-	14
		Social taboo of menstruation	-	-	13	-	-	13
	Pain	Pain that is variable, intense and disabling	17	22	9	10	13	71
		Uncertainty, stress and fear	4	4	2	4	7	21
Body and mood Changes		Bodily changes and self-perception	5	13	-	1	1	20
		Changing how they dress	2	10	-	-	-	12
		Mood swings	7	11	13	7	8	46
		Lack of energy and disruption of daily activities	23	21	14	17	16	91
		Increased sensitivity/irritability with others	7	11	13	6	12	49
Seeking a safe environment	A safe environment	Feeling understood	9	12	2	5	2	30
		To be able to speak freely about dysmenorrhea	2	4	-	1	-	7
		People close to you see the consequences of pain	3	7	-	2	2	14
	An unsafe environment	When others disregard pain	2	5	-	3	3	13
		Women who understand it when they experience it	8	6	-	4	5	23
		Women who haven't had a period in a long time	-	2	-	1	-	3
		Unknown environments	1	4	-	2	1	8
	Key issues regarding security	A person close to you may or may not belong to the safe environment	3	3	2	2	1	11
		Men may or may not understand dysmenorrhea/pain	2	12	1	1	2	18
		Belonging to the safe environment is not a matter of gender, but of empathy	3	9	-	1	4	17
		Concealing it in the practice/work environment	-	9	1	-	1	11
